# The Interconnection among Post-Traumatic Stress Disorder, Sexual Dysfunction, and Severe Postpartum Hemorrhage

**DOI:** 10.1192/j.eurpsy.2026.11999

**Published:** 2026-07-31

**Authors:** N. Smaoui, A. Jedidi, S. Omri, R. Feki, H. Hkim, I. Gassara, M. Maalej, M. Maalej, N. Charfi, L. Zouari

**Affiliations:** 1 Psychiatry C; 2 Gynecology-Obstetrics Department, Hedi Chaker Hospital, Sfax, Tunisia

## Abstract

**Introduction:**

Severe postpartum hemorrhage (PPH) can be a life-threatening obstetric complication with enduring consequences beyond the immediate physical risks. Increasing evidence suggests that PPH may trigger psychological trauma, notably post-traumatic stress disorder (PTSD), which itself can negatively affect sexual health. Exploring the interconnection between PTSD and sexual dysfunction in women exposed to severe PPH is crucial for understanding the full spectrum of its impact and guiding comprehensive postnatal care.

**Objectives:**

The aim of this study was to investigate the relationship between post-traumatic stress disorder (PTSD), sexual dysfunction, and severe postpartum hemorrhage (PPH).

**Methods:**

A cross-sectional, descriptive, and analytical study was conducted between January and December 2024 among 49 women who experienced severe PPH between 2017 and 2023 at the Gynecology-Obstetrics Department of Hedi Chaker University Hospital in Sfax.

PTSD was assessed using the Post-Traumatic Stress Disorder Checklist for DSM-5 (PCL-5), with a diagnostic threshold set at a total score ≥ 31. Sexual function was measured using the Female Sexual Function Index (FSFI), with a total score ≤ 26.55 indicating sexual dysfunction. Statistical analysis was performed using SPSS-26 software.

**Results:**

The mean PCL-5 score was 31.81 ± 18.54 (range: 4–71), with 44.9% of participants (22/49) meeting diagnostic criteria for PTSD.

The mean FSFI total score was 19.3 ± 8.15 (range: 0–34.5), with sexual dysfunction observed in 79.6% of participants (n = 39).

A highly significant association was observed between PTSD and sexual dysfunction. Participants with PTSD were significantly more likely to report sexual dysfunction than those without PTSD (44.9% vs 34.7%; p = 0.001).

**Conclusions:**

Severe PPH was associated with a high risk of PTSD, which in turn appears to contribute to marked sexual dysfunction. These findings underscore the importance of targeted PTSD screening in women who have experienced severe PPH to enable early, specialized interventions aimed at preserving both sexual and mental health.

**Disclosure of Interest:**

None Declared

